# *Octopus minor* Antimicrobial Peptide-Loaded Chitosan Nanoparticles Accelerate Dermal Wound Healing

**DOI:** 10.3390/ijms26199701

**Published:** 2025-10-05

**Authors:** Mawalle Kankanamge Hasitha Madhawa Dias, Shan Lakmal Edirisinghe, Mahanama De Zoysa, Ilson Whang

**Affiliations:** 1 College of Veterinary Medicine, Chungnam National University, Daejeon 34134, Republic of Korea; hasithadiasm17636@gmail.com (M.K.H.M.D.); shanlakmal09011@gmail.com (S.L.E.); 2 National Marine Biodiversity Institute of Korea (MABIK), 75, Seochun-gun 33662, Republic of Korea

**Keywords:** wound healing, Octominin, AMPs, nanoencapsulation, nanoparticles, zebrafish

## Abstract

Octominin is a peptide derived from the *Octopus minor* defense protein, which has shown antimicrobial and immunomodulatory properties. The present study describes the efficacy of Octominin-encapsulated chitosan (CN) nanoparticles (Octominin-CNPs) on in vitro and dermal wound healing in zebrafish. Initial viability analysis revealed there was no significant toxicity of Octominin-CNPs up to 200 μg/mL in human dermal fibroblast (HDF) cells and in zebrafish larvae (up to 50 μg/mL). Moreover, the potential wound healing activity of Octominin-CNPs was observed using the cell-scratch assay. In the in vivo study, wounded adult zebrafish were applied with the appropriate treatment (PBS, CNPs, Octominin, and Octominin-CNPs) 20 μg/wound/fish as a topical application at 0, 2, and 4 days post-wounding (dpw) while photographs of each wound site were taken at 2, 4, 7, 10, 14, and 21 dpw, and surface area was measured using ImageJ software (Ver. 1.8.0, NIH, Bethesda, MD, USA) to calculate the wound healing percentage (WHP) and wound healing rate (WHR). From the observed results, at 4 dpw, all treatments showed a negative impact on wound healing, where the lowest WHR and the WHP were given by the negative control (NC) until the 14th day. After 7 dpw, all fish except the NC showed increased wound healing activity. Compared to the Octominin, the Octominin-CNPs showed higher activity, which was at its peak on 21 dpw. Furthermore, Octominin-CNPs suppressed the expression of pro-inflammatory cytokine and chemokine mRNA expression with increased wound healing efficacy, and tissue repair compared to the Octominin-alone-treated fish at 7 dpw. Together, the observed results give insights into the use of nanoencapsulation as a means of drug delivery, especially for small peptides.

## 1. Introduction

Wound healing is a highly complex biological process that occurs as a result of tissue damage, involving multicellular coordination events leading to tissue repair and remodeling [1]. In mammals, wound healing begins with hemostasis, followed by inflammation, cellular proliferation, and dermal remodeling, leading to complete repair [1,2]. The cumulative effort of various cell types and molecular mediator-associated complex mechanisms maintains the wound healing process in a timely manner, keeping the host safe [3]. However, due to a number of intrinsic (immune deficiency, diabetes-like diseases, malnourishment, etc.) and extrinsic factors (relative humidity, temperature, atmospheric pollutants, etc.), this complex biological system can lose its balance, leading to a state of chronic infection, secondary disease progression, and dysregulation in tissue repair [1,2,4]. Therefore, it is of utmost importance to minimize the barriers that hinder the uninterrupted wound healing process.

Antimicrobial peptides (AMPs) have emerged as one of the prominent small molecules currently utilized by various industries due to their versatility of usage and activity, as well as an alternative to multidrug resistance development [5,6]. Also known as host defense peptides, AMPs’ modes of action vary between membrane-disruptive and non-membrane-disruptive, affecting different components of the target microorganism, minimizing its growth and reproduction cycle [6,7]. Membrane-disruptive AMPs are believed to interact with negatively charged phospholipid membranes, facilitating transmembrane pore formation, and damaging and increasing membrane permeability, leading to cell lysis and cell death [8]. The non-membrane-disruptive AMPs freely cross into the cell, interacting with negatively charged macromolecules (DNA and RNA), directly affecting protein synthesis and enzyme activity, destroying cellular structures [6,8]. Although AMPs have been studied for decades, only a handful have been developed from laboratory research to clinical applications (NP213/Novexatin^®^ against onychomycosis fungi, NBI 226/Omiganan against human tumor virus-induced genital lesions), mainly because of their natural vulnerability to enzymatic degradation, toxicity, and pharmacokinetic constraints [9]. However, molecular mechanisms of potential AMPs from research studies hold vital information that enables them to be commercialized for clinical use, which the majority of the studies lack. Due to their small molecular structure and cationic charge, AMPs have the advantage of entering the target cell quickly, initiating the anti-microbial effects. Therefore, AMPs are studied as alternative therapeutics in wound healing studies through topical applications [10]. However, certain AMPs are highly soluble in water, limiting the time of contact with the wound for aquatic organisms. Furthermore, direct use of AMPs in topical applications limits their effectiveness and activity due to adverse environmental and host conditions, making it a further challenge [10]. Therefore, stable, prolonged delivery and increased efficiency should be achieved when using AMPs as potential wound healing agents.

The incorporation of nanotechnology has become one of the rising trends in the 21st century, pioneering a vast spectrum of industries from agriculture to medicine, with limitless potential applications [11]. Vesicular-based AMP delivery, including liposomes, micelles, liquid crystalline systems, meso-porous nanoparticles, nanofibers, carbon nanotubes, polymeric nanoparticles, and microspheres, has been widely studied for its applications to overcome limitations of decreased sensitivity and cytotoxicity of AMPs, while controlling the release to the target site [12]. Nanoencapsulation is a method utilized to treat diseases via oral administration and topical application of therapeutics [13]. Various studies have identified chitosan (CN) as one of the biodegradable, bio-compatible, non-toxic, feasible materials that can be utilized for developing nanoencapsulation-based therapeutic agents [14,15,16]. Moreover, CN itself has the ability to protect the active AMPs from degradation while anchoring to exposed mucus membrane layers, releasing the encapsulated AMP steadily for maximum efficiency [17].

Octominin is one of the known AMPs that is synthetically developed from the *Octopus minor* defense protein 3, and has been identified as a potential candidate against inflammatory responses as well [18,19]. The current study focuses on the wound healing potential of Octominin, which has never been reported, as well as the efficacy of encapsulating Octominin in chitosan (CN) to form Octominin-encapsulated CN nanoparticles (Octominin-CNPs), to compare the activity of use and its potential as a future wound healing therapy.

## 2. Results

### 2.1. Toxicity of Octominin-CNPs In Vitro and In Vivo

**Figure 1:**
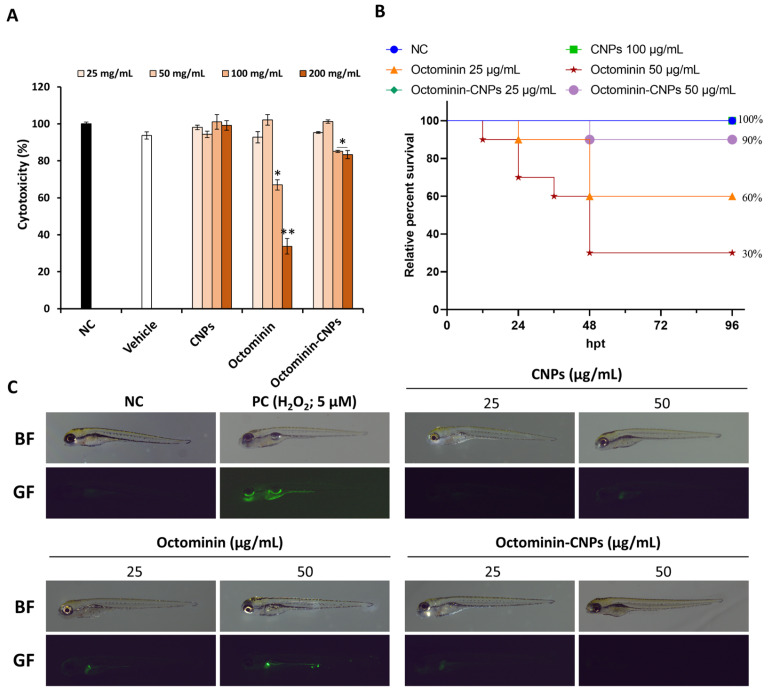
Toxicity of Octominin-CNPs (**A**). Cytotoxicity was assessed in Octominin-CNPs pretreated HDF cells using the Cellrix^®^ viability assay kit (Data are presented as means ± SEM in triplicates). (**B**) In vivo toxicity was evaluated in zebrafish larvae (60 hpf). After treating larvae with Octominin-CNPs (n = 10/treatment concentration), mortality was monitored till 96 hpt. (**C**) ROS generation was analyzed in Octominin-CNPs pretreated larvae (n = 3/treatment) using DCFHDA stain (5 µg/mL). Hydrogen peroxide (H_2_O_2_; 5 µM) was used as the positive control. (* = *p* < 0.05, ** = *p* < 0.01) (GF—green fluorescence; BF—bright field).

The cytotoxicity of Octominin-CNPs was evaluated to determine the optimum concentration that can be safely administered into human dermal fibroblast (HDF) cells. As shown in Figure 1A, treatment of HDF cells with Octominin-CNPs up to 50 µg/mL did not result in significant cytotoxicity, whereas Octominin alone exhibited considerable toxicity at higher concentrations. The IC_50_ value of Octominin was 99.43 μg/mL, while the IC_50_ value of Octominin-CNPs was >200 μg/mL. In vivo toxicity of Octominin-CNPs was evaluated using 60 h post-fertilization (hpf) zebrafish larvae (Figure 1B). Larvae in the negative control (NC) and CN nanoparticles (CNPs; 100 µg/mL)-treated groups had no mortality until 96 h post-treatment (hpt). In contrast, Octominin-treated larvae showed 60% and 30% survival at 25 and 50 µg/mL concentrations, respectively. Octominin-CNPs (50 µg/mL)-treated larvae showed 90% survival at 96 hpt. Reactive oxygen species (ROS) generation in pretreated zebrafish larvae demonstrated comparatively higher green fluorescence for the Octominin-treated group than for the Octominin-CNPs-treated group at both concentrations (Figure 1C). Based on these observations, the Octominin-CNPs concentration range 25–50 μg/mL was selected as the optimum range for all treatments in this study.

### 2.2. In Vitro Wound Healing Activity of Octominin-CNPs

**Figure 2:**
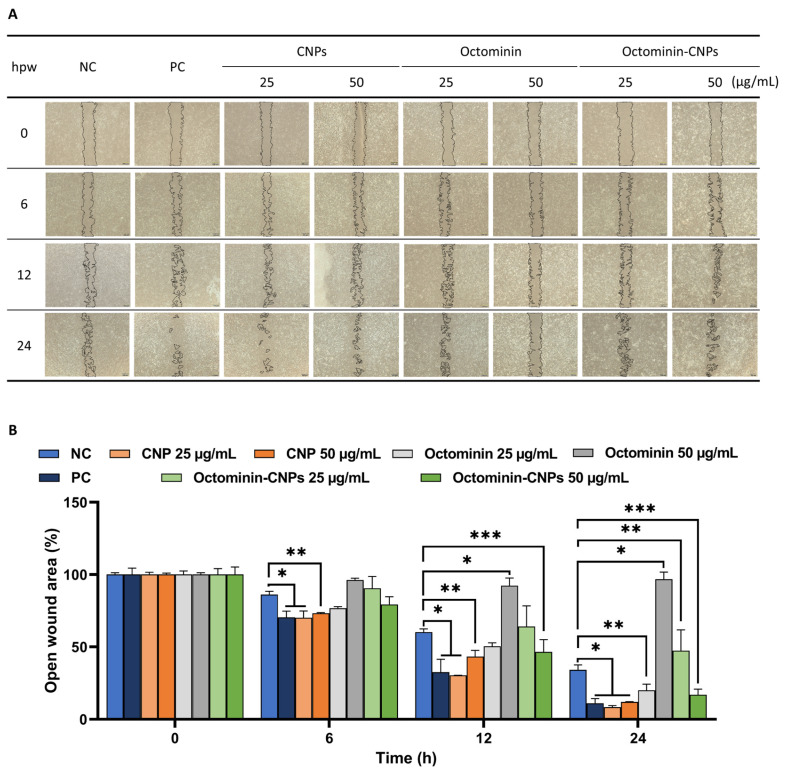
In vitro wound healing efficacy of Octominin-CNPs. (**A**). Representative images of the cell scratch assay performed using HDF cells to assess the wound healing effect of Octominin-CNPs. Cell-free area reduction was observed and compared against the NC group at 0, 6, 12, and 24 h post-wounding (hpw) (scale bar = 100 µm). (**B**). Quantification of the cell scratch assay showing the open wound area (%), calculated relative to the NC group at each time point using the ImageJ software. Triplicates were treated and averaged to ensure repeatability, and statistical significance was measured at *p* < 0.05 = *, *p* < 0.01 = **, and *p* < 0.001 = ***. Data are presented as means ± SEM. (PC—Low serum fibroblast basal medium).

As depicted in Figure 2A, cellular mono-layer replenishment gradually increased in all treatment groups over time. The highest cell migration was observed in the CNPs and the positive control (PC)-treated groups. Notably, cells treated with Octominin-CNPs showed a greater cell migration than those treated with Octominin, effectively filling the free space. Analysis of the images revealed that Octominin-CNPs reduced the cell-free gap in a concentration-dependent manner compared to cells treated with Octominin alone at all time points (Figure 2B). At 24 hpt, the smallest wound area was observed in CNPs-treated cells (25 µg/mL; 8.33% and 50 µg/mL; 12.02%), while the PC showed 11.13% of the open wound area. Interestingly, Octominin-CNPs at 50 μg/mL demonstrated wound closure comparable to the PC, highlighting its wound healing properties and the effective delivery of the encapsulated Octominin.

### 2.3. Enhanced Wound Healing Effects in Octominin-CNPs-Treated Zebrafish

**Figure 3:**
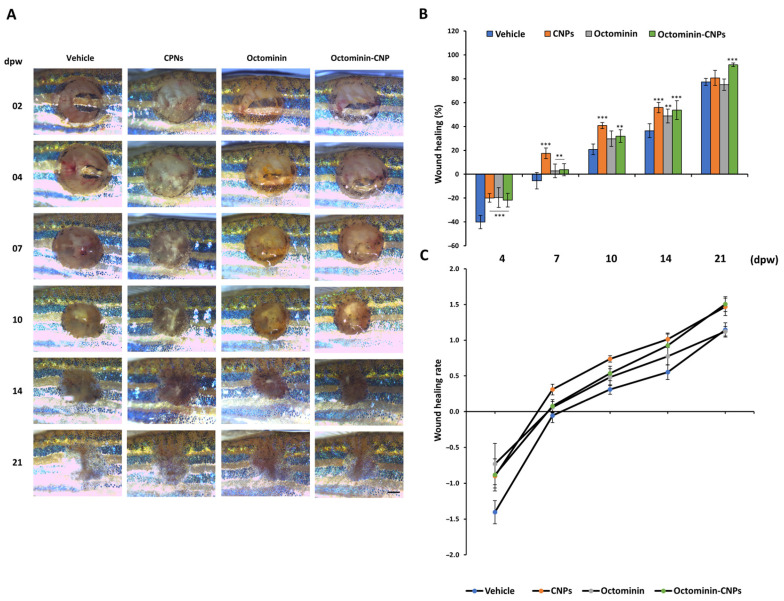
In vivo wound healing activity of Octominin-CNPs. (**A**). Representative images of dermal wound healing from 2 to 21 days post-wounding (dpw) (individually maintained 5 fish/treatment) (scale bar = 1 mm). Wound closure was monitored from 2 to 21 dpw, and wound area reduction was quantified relative to wound size at 2 dpw to calculate (**B**) wound healing percentage (WHP), and (**C**) wound healing rate (WHR) at 4, 7, 10, 14, and 21 dpw. Data are presented as means ± SEM. (** = *p* < 0.01, *** = *p* < 0.001) (dpw—days post-wounding).

The in vivo wound healing study was conducted using an adult zebrafish model with a mechanically initiated wound (3 mm diameter biopsy punch). Microscopic observations showed an initial increase in the wound area at 4 days post-wounding (dpw) compared to the initial images, likely reflecting an inflammatory response and possible infection due to exposure of the wound to the surrounding environment. Over time, the open wound areas in all groups gradually decreased, and at 21 dpw, most wounds had largely recovered with no distinguishable wound margins and increased pigmentation (Figure 3A). Moreover, compared to the vehicle group, the CNPs and Octominin-CNPs groups showed rapid wound closure, as indicated by the visible reduction in the free wound margins in a time-dependent manner. This was further confirmed by evaluating the wound healing percentage (WHP) and wound healing rate (WHR) (Figure 3B,C), where Octominin-CNPs demonstrated a significantly higher (*p* < 0.05) WHP at 21 dpw (91.73 ± 1.56%) compared to the vehicle group (77.35 ± 2.88%). The highest WHR was also recorded at 21 dpw in the Octominin-CNPs (1.50 ± 0.10) compared to other treatment groups.

### 2.4. Re-Epithelialization and Tissue Regeneration of Octominin-CNPs-Treated Zebrafish

**Figure 4:**
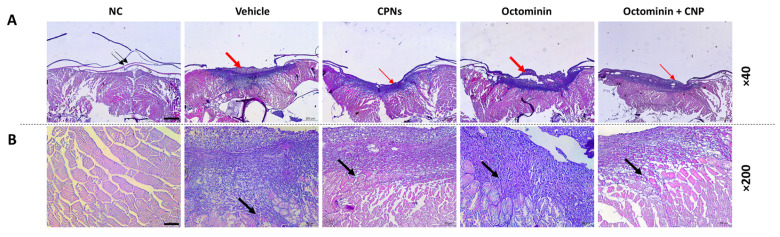
Representative microscopic images from histological analysis of Octominin-CNPs mediated wound healing activity. Wound muscles (n = 3) isolated at 7 dpw were stained with H&E and examined to evaluate (**A**) epidermis and muscle tissue (×40; scale bar = 200 µm) and (**B**) damaged muscle tissue (×200; scale bar = 50 µm). Thin black arrows indicate the typical epidermis with scales in the unwounded negative control (NC) group. Thick black arrows denote infiltration of inflammatory cells in damaged muscle tissue. The thin red arrows highlight reduced granular tissue formation, whereas thick red arrows indicate extensive granulation tissue formation with multiple cell layers and neo-epidermis.

To further confirm the wound healing effect of Octominin-CNPs in zebrafish, muscle tissues isolated from wound sites were subjected to hematoxylin and eosin (H&E) staining and examined under a microscope (Figure 4). Unwounded zebrafish skin displayed a multilayered epidermis that had a thin fibroblast layer, and the outermost scales, which overlap adjacent scales (Figure 4A; NC). After wounding, the entire structure, including scales and fibroblasts, was damaged, exposing the underlying muscle tissue. At 7 dpw, the vehicle group showed pronounced granulation and extensive inflammatory cell infiltration (Figure 4A,B). Compared to the vehicle group, Octominin-CNPs-treated zebrafish muscle tissues exhibited rapid re-epithelialization and lower inflammatory cell infiltration (blue staining), indicating progression to later stages of wound healing. Moreover, compared to Octominin treatment, both CNPs and Octominin-CNPs promoted higher re-epithelialization and tissue regeneration, resulting in a more organized muscle tissue structure (Figure 4A,B). These findings were further supported by a semi-quantitative analysis of tissue samples at 7 dpw (Figure S1A,B). Overall, CNPs and Octominin-CNPs significantly reduced inflammation, tissue necrosis, and granulation while enhancing re-epithelialization, thereby accelerating wound healing compared to both the vehicle- and Octominin-treated groups.

### 2.5. Octominin-CNPs Treatment Reduced the Pro-Inflammatory Gene Markers in Zebrafish

**Figure 5:**
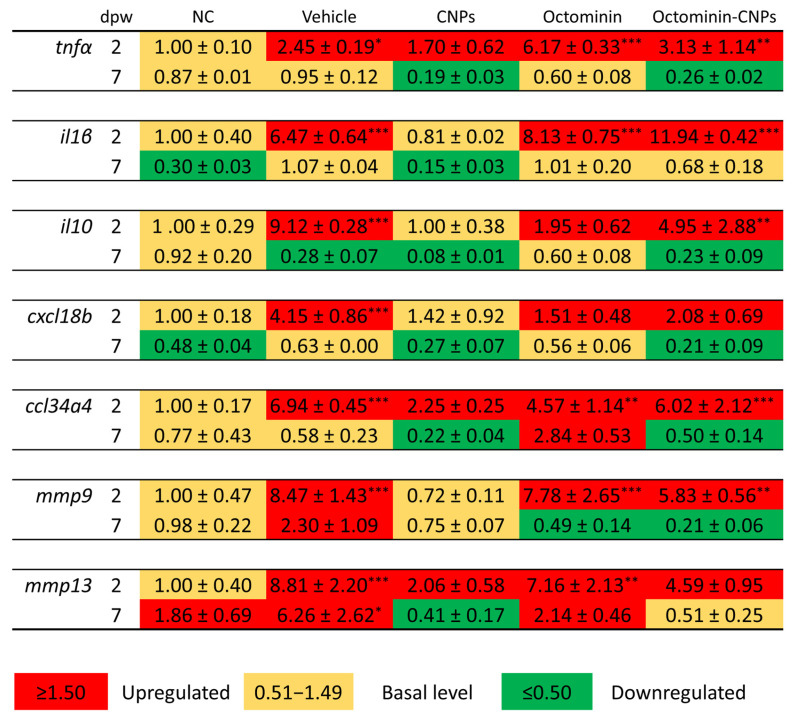
Heat map summarizing transcriptional regulation during in vivo wound healing upon Octominin-CNPs treatment. Wound muscle tissues (2 tissues/replicate; 3 replicates/treatment/time point) from zebrafish at 2 and 7 dpw were isolated, and gene expression levels were quantified from the prepared cDNA using the 2^−ΔΔCT^ method. The basal expression level of the control group was normalized to 1-fold. Data are presented as mean relative expression folds ± SEM with triplicates. Statistical significance was determined at *p* < 0.05 = *, *p* < 0.01 = **, and *p* < 0.001 = ***.

To further validate the potential in vivo wound healing activity of Octominin-CNPs, zebrafish muscle tissues were analyzed for the transcriptional expression of selected genes (Figure 5). These genes were categorized into three groups: inflammatory cytokines [tumor necrosis factor (*tnf*)*α*, interleukin (*il*)*1β*, and *il10*], chemokines [chemokine (C-X-C motif) ligand 18b (*cxcl18b*) and chemokine (C-C motif) ligand 34a, duplicate 4 (*ccl34a4*)], and tissue remodeling/repair genes [matrix metalloproteinase (*mmp*)*9* and *mmp13*]. The pro-inflammatory cytokines *tnfα* and *il1β* were upregulated across all groups, except *il1β* in the CNPs group at 2 dpw. The highest expression was observed in the Octominin-CNPs group (*il1β*; 11.94 ± 0.42-fold). Notably, anti-inflammatory cytokine *il10* showed a similar expression pattern to that of *il1β* at the same time point. Both chemokines (*cxcl18b* and *ccl34a4*) were upregulated in the wounded vehicle, Octominin, and Octominin-CNPs-treated groups, where CNPs-treated *ccl34a4* expression was also upregulated (2.25 ± 0.25-fold). Apart from the NC and CNPs group, *mmp9* expression was upregulated in all other groups, while *mmp13* was at a basal level for the NC group.

At 7 dpw, relative expression folds for selected cytokines, chemokines, and *mmp9* declined, while *mmp13* expression in the unwounded NC group upregulated (1.86 ± 0.69-fold). Overall, the mechanically induced dermal wound in zebrafish led to an upregulation of all genes in most groups (vehicle, Octominin, and Octominin-CNPs) compared to the NC group, which is expected due to the stress and inflammation triggered by wounding. However, this upregulation was markedly lower in the Octominin-CNPs group compared to the Octominin group, suggesting the potential advantage of delivering Octominin in an encapsulated form. Furthermore, the downregulation of pro-inflammatory cytokines (*tnfɑ* and *il1β*) and chemokines (*cxcl18b* and *ccl34a4*) suggests a reduction in wound-induced inflammation.

## 3. Discussion

The use of AMPs has recently increased in therapeutic applications against a variety of pathogenic microorganisms and parasites due to their versatility for use in different industries, such as food, medicine, animal rearing, agriculture, and aquaculture, to mitigate the concerns of the overuse of antibiotics [20,21]. Octominin is one such candidate that has been synthetically developed by selecting an amino acid fragment of the defense protein 3 from the *Octopus minor* transcriptome database and modifying it to be used as a potential anti-microbial agent [18]. Much research has been conducted to elucidate the anti-bacterial, anti-fungal, and anti-inflammatory activity of Octominin [18,19,22]. Therefore, the potential application of Octominin as a therapeutic agent in wound healing could be a viable source due to its proven anti-microbial and anti-inflammatory activity.

One of the key challenges to overcome with the use of AMPs is their low stability in in vivo conditions. Interactions with the host immune cells, host temperature, adverse pH conditions, and enzymes may lead to proteolytic degradation of the AMPs, reducing their true activity when tested in living organisms [5]. To overcome this obstacle, nanoencapsulation of Octominin is practiced in the present study. CN is an amino polysaccharide that is highly researched for having its characteristic properties, such as biodegradability, low toxicity, and bio-compatibility [16]. Moreover, researchers have pioneered nanoencapsulation techniques using CN as a base, due to its ability to interact with surrounding chemicals and form gels, films, and nanocapsules [23]. However, one of the major downsides when it comes to the strategy of nanoencapsulating a cationic AMP like Octominin is that it limits the encapsulation efficiency, due to the positive charge of CN. Therefore, to minimize this unfavorable effect, the use of a negatively charged cross-linker during the ionotropic gelation process is an ideal solution [22]. Previous studies have been conducted to better understand and optimize this phenomenon, where a cationic amphiphilic AMP, temporin B, and substrate renin were successfully loaded into CN while using sodium tripolyphosphate as the negative charge cross-linker [15,17]. Similarly, in the present study, Carboxymethyl-chitosan (CMC) was opted as the anionic cross-linker to prepare the Octominin-CNPs that have the potential to improve stability and solubility with additional wound healing activity [24,25]. In a previous study, the highest encapsulation efficiency (96.49%) and loading capacity (40.20%) for Octominin-CNPs were given by mixing CN: CMC: Octominin at a ratio of 0.4:2:1 [22]. Furthermore, it showed potent antimicrobial activity against *Acinetobacter baumannii*, *Candida albicans*, together with anti-biofilm activity as well [22]. Hence, in the present study, we implemented the same ratio for preparing Octominin-CNPs, hypothesizing that the physicochemical and biological properties of the Octominin-CNPs remained unchanged.

Cytotoxicity analysis revealed that Octominin-CNP was significantly less toxic compared to Octominin alone in HDF cells up to the concentrations of 200 μg/mL. A significant viability reduction (*p* < 0.05) in a concentration-dependent manner was observed for Octominin above 50 μg/mL. Furthermore, Jayathilaka et al. observed similar results in human embryonic kidney 293 cells, emphasizing that it is safer to treat the encapsulated Octominin at higher concentrations, in vitro [22]. Moreover, non-toxic, biologically compatible CN and CMC, used for nanoencapsulation, may indirectly minimize the cellular toxicity by slowly releasing Octominin and maintaining cellular homeostasis [14,24]. This was further observed from the zebrafish larvae survival analysis and ROS determination, where Octominin-CNPs (50 μg/mL) showed less toxicity and ROS generation compared to the Octominin-only-treated group [22]. This further confirms the potential implementation of Octominin-CNPs in cellular and animal models as an AMP delivery system.

Apart from its obvious activity against harmful microbes, wound healing is another aspect that AMPs are known for [8]. Studies revealed that human-derived LL37 and S100 AMPs reduce collagen production in keloid tissues, minimizing fibroblast proliferation and enhancing the wound healing effect [8]. Furthermore, recombinant LL37 was reported to promote wound healing by accelerated endothelial cell proliferation and migration, leading to increased vascularization and re-epithelialization in trauma mouse models [26]. A study conducted by Yan et al. reported AMP YD’s anti-fibrotic activity, mediated by reducing inflammation via a miR-155–Casp12–NF-κB pathway axis, suggesting a hand-in-hand involvement of regulating inflammation and wound healing [27]. Moreover, Sanjeewa et al. observed that there were anti-inflammatory effects of Octominin when treated to an LPS-stimulated Raw 264.7 murine macrophage cell line, implying its potential cell proliferative effect [19]. Furthermore, various reports confirm that the materials used for the encapsulation process alone (CN and CMC) possess wound healing activity and have been used for applications in wound dressing in modern medicine [14,16,24]. Results observed in the present study (Figure 2) showed increased cellular proliferation and migration when the cell scratch assay was conducted with Octominin-CNPs. Interestingly, Octominin-CNPs’ cellular proliferation activity was comparatively higher than that of the Octominin-only-treated cell group, which can be due to CN and CMC being proven wound healing active agents [14,24]. From this, it is apparent that the encapsulation of Octominin with CN using CMC as a cross-linker further improved the cellular proliferation and migration, reducing the time to completely cover the cell layer.

When an injury occurs, to avoid secondary infection by exposure to pathogens and hazardous materials, skin regenerates itself, rapidly acquiring barrier function. This phenomenon in adult mammals involves a multi-step complex process that includes blood clotting, inflammatory reactions, re-epithelialization, vascularization, and tissue regeneration, leading to tissue maturation [28]. However, zebrafish are widely adapted as a model animal to study mechanisms and biological pathways involved in wound healing, without compromising the major steps involved in mammalian wound healing [28,29].

Compared to wound healing in mammals, zebrafish lack the hemostatic phase, where blood clotting occurs. This is replaced with re-epithelialization after immediate wounding [29]. During this phase, the bottom section of the wound will be rapidly filled with connective tissues, forming the base. Then, macrophage and neutrophil infiltration happen to minimize and survive the inflammatory phase, which lasts for 1–5 dpw. After day 6, the granulation occurs, and with time, dermal thickness increases, which helps to regrow the lost scales, leading to fully repaired tissue by the 30th day [28]. Phenotypically, this was observed from 2 dpw to 21 dpw, where a gradual reduction in the open wound area was observed with partial to full pigmentation. Initial WHR and WHP were negative, due to a slight increment of the open wound area during the initial inflammatory stage of the wound healing process, which was similarly observed in a previous study [30]. Even though CNPs had comparatively higher WHP and WHR until 10 dpw, Octominin-CNPs showed the highest WHR and WHP at 21 dpw with greater pigmentation, highlighting their superior wound healing potential. However, identifying the actual wound margin during the early and later stages of wound healing, due to the early onset of inflammation and heavy pigmentation, is a limitation that can impact the outcome.

Although microscopic monitoring of dermal wounds provides valuable insights into wound filling and closure, tissue remodeling, and healing, it lacks the important histopathological changes that occur during wound healing [30]. Furthermore, it provides additional information at a cellular level, minimizing the limitations of microscopic wound monitoring. With the observed results, even at 7 dpw, the re-epithelialization was still highly present in the wounded vehicle group and the Octominin-treated group, with high inflammatory cell infiltration. However, in the Octominin-CNPs-treated group, moderate granulation was present with minimum re-epithelialization and less inflammatory cell infiltration. The semi-quantitative analysis of the wound tissues showed similar results. A study conducted by Edirisinghe et al. observed similar findings at 7 dpw with less granulation, inflammatory cell infiltration, and higher tissue remodeling when treated with *Spirulina maxima*-based pectin (SmP) compared to the wounded vehicle group [31]. Overall, this suggests that the Octominin-CNPs treatment accelerated the wound healing process, minimizing bacterial infection-stimulated inflammation, simultaneously healing the wound compared to the Octominin-treated group.

To further explain the mechanism of tissue regeneration and repair with the treatment of Octominin-CNPs, prominent molecular gene markers were analyzed using quantitative real-time polymerase chain reaction (qRT-PCR). Pro-inflammatory cytokine expression is one of the key markers of an ongoing infection within the body [32]. The release of *Tnfα* and Il1β can upregulate the secretion of pro-inflammatory proteins and further stimulate immune cells to differentiate, which maintains the inflammatory response [33]. Simultaneously, chemokines such as *cxcl18b* and *ccl34a*, which are known inflammatory markers in zebrafish, also increase expression during an ongoing infection [34]. Observing the results, it is clear that with the treatment of Octominin-CNPs, there was a noticeable reduction in *tnfα*, *il1β*, and *il10* levels, implying that the ongoing inflammation was minimized rapidly compared to other treatments. This was further confirmed with the reduction in *cxcl18b* and *ccl34a* compared to the vehicle group. Expression of *mmp* genes in the skin is associated with tissue repair and regeneration. Out of those, *mmp9* and *mmp13* are involved in cell migration, tissue granulation, and remodeling of the scar tissue [31]. In the present study, both genes were upregulated in Octominin-CNPs and Octominin alone treatments, although in both instances, it was lower compared to the vehicle at 2 dpw. At 7 dpw, the expression of both genes was downregulated. However, the level of downregulation of both genes was much higher in the Octominin-CNPs-treated group compared to other groups. This suggests that Octominin-CNPs accelerate tissue regeneration, potentially contributing to the observed gene downregulation. A similar pattern of relative gene expression was observed in a previous publication, when zebrafish were fed with a multi-strain yeast fraction (MsYF) [30]. Furthermore, encapsulating AMP Octominin with CN further increases the chance of adhering to the mucosal layer of the open wound, keeping the active ingredient from degradation or washout [16]. This phenomenon, coupled with the slow and steady delivery of Octominin to the wound site, may help to minimize inflammation, as well as microbial infections, while accelerating the healing mechanisms. Given its reported antimicrobial and anti-inflammatory activities, our findings highlight the efficacy of Octominin-loaded nanoparticles (Octominin-CNPs) as a potential therapeutic for dermal wounds.

## 4. Materials and Methods

### 4.1. Raw Materials, Chemicals, and Reagents

HDF (ATCC PCS-201-010™) cells and PCS-201-030™ (Fibroblast basal medium) were purchased from ATCC (Livingston, MT, USA). Fetal bovine serum (FBS) and a mixture of antibiotics were obtained from Welgene (Gyeongsan-si, Republic of Korea). Disposable biopsy punches (3 mm) were obtained from Kai Industries (Oyana, Japan). Primers for the qRT-PCR were designed and purchased from BiONEER (Daejeon, Republic of Korea). TB Green Premix Ex Taq II was purchased from TaKaRa (Shiga, Japan). Tricaine, 0.5 M ethylenediaminetetraacetic acid (EDTA), acetic acid, and 10% neutral buffered formalin were purchased from Sigma-Aldrich (St. Louis, MO, USA). Minisart^®^ syringe filters (0.1 μm diameter) were bought from Sartorius Lab Instruments (Goettingen, Germany). All other chemicals and reagents used for the study were of the highest analytical grade.

### 4.2. Preparation of Octominin-Encapsulated Chitosan Nanoparticles

The designing and synthesis of *Octopus minor*-derived Octominin AMP was performed according to a previous methodology [18]. Octominin-CNPs were prepared following the ionotropic gelation method described in a previous manuscript [22]. Briefly, a CN solution (1 mg/mL; pH 5) was prepared in a 1% (*v*/*v*) acetic acid solution (pH 5), and simultaneously, a CMC solution (1 mg/mL; pH 7.4) was prepared in distilled water. After syringe-filtering both solutions using a 0.1 μm Minisart^®^ syringe filter, Octominin solution (1 mg/mL; 1 mL) was mixed with 2 mL of CMC while balancing the volume with distilled water. Continuous agitation was conducted for 40 min using a magnetic stirrer, and 0.4 mL of CN solution was gradually added without discontinuing the agitation (CMC:Octominin:CN = 2:1:0.4). After 1.5 h of additional mixing, the resulting mixture was centrifuged at 12,000 rpm for 30 min at 4 °C to collect the encapsulated nanoparticles. Octominin-CNPs were finally separated from the supernatant and suspended in 1× phosphate-buffered saline (PBS; pH 7.4) for further use.

### 4.3. Cells and Zebrafish Maintenance

HDF cells were maintained in a fibroblast basal medium supplemented with 2% FBS in an atmospherically controlled environment provided with 5% CO_2_ at 37 °C. Periodical subculture was practiced three times per week until 90% confluency was reached. Adult zebrafish used for experiments were reared following the methods and conditions described in a previous publication [31]. The light and dark cycles were 14 h and 10 h, respectively, and the water temperature was kept at 28 °C. In vivo experiments used in the study were under the approved regulations and guidelines provided by the Animal Ethics Committee of Chungnam National University.

### 4.4. In Vitro and In Vivo Toxicity of Octominin-CNPs

Cellular toxicity of Octominin-CNPs was evaluated using Cellrix^®^ Viability Assay Kit (MediFab, Geumcheon, Republic of Korea), following the recommended protocol with minor modifications [35]. Briefly, cells were seeded in 96-well plates (2 × 10^4^ cells/mL/well), where 90 μL of the cell suspension was transferred to each well and incubated at 37 °C, supplied with 5% CO_2_. After 12 h, 10 μL of each treatment (n = 3, per treatment) was applied at different concentrations and incubated for an additional 24 h, where the NC group was treated with 1× PBS. Following the incubation period, 10 μL of Cellrix^®^ Viability Assay reagent was added to each well and kept inside the same incubator for 2.5 h to allow color development. The absorbance was measured at 450 nm using a iMark microplate reader (BIO RAD, Tokyo, Japan).

Zebrafish larvae toxicity was determined by following the procedure of a previous publication [36]. Healthy larvae (60 hpf) were treated with Octominin, CN, and Octominin-CNPs (0, 25, 50, 100 µg/mL; n = 10/concentration of treatment), and mortality was observed for 96 hpt with 12 h intervals. At 96 hpt, three larvae from each treatment group were used to determine the oxidative stress by staining with 2′7′dichlorodihydro-fluorescein diacetate (DCFHDA) (Sigma-Aldrich, St. Louis, MO, USA) (5 μg/mL) for ROS. Images were taken using a stereo microscope (Leica S8 APO, Leica Microsystems GmbH, Wetzlar, Germany), equipped with a Stereo Microscope Fluorescence Adapter (SFA) system (NIGHTSEA, Hatfield, PA, USA).

### 4.5. In Vitro Wound Healing Activity Analysis

The wound healing activity of Octominin-CNPs was evaluated using HDF cells, following a previously described method [31]. HDF cell suspension was seeded in a culture insert 2-well plate, 70 μL per well at 2 × 10^5^ cells/mL density (n = 3, per treatment). After 24 h of incubation, a confluent cell monolayer was formed, and the inserts were carefully removed to form a gap that was free of cells, resembling a wound condition. Serum-free media was transferred for each dish (2 mL per well), containing Octominin-CNPs at different concentrations (25 and 50 µg/mL). For the NC group, 1× PBS was used, and for the PC group, 2% FBS-supplemented fibroblast basal medium was used. Images of the cell-free gaps were captured using an inverted light microscope (Leica^®^ DMi8, Leica Microsystems GmbH, Wetzlar, Germany) at 0, 6, 12, and 24 h intervals to observe the cell migration rate. ImageJ software (Ver. 1.8.0, NIH, Bethesda, MD, USA) was used for the image quantification, and normalization was performed using 0 h cell-free gap values.

### 4.6. Dermal Wound Initiation and Topical Application of the Treatment on Adult Zebrafish

Uniform-sized (average body weight 0.45 ± 0.05 g) and aged (four months old) zebrafish were selected for the experiment. Selected zebrafish were divided into five groups (25 fish/group): (1). NC (no wound); (2). vehicle (wounded and treated with 1 × PBS); (3). CNPs (wounded and treated with CNPs); (4). Octominin (wounded and treated with Octominin peptide), and (5). Octominin-CNPs (wounded and treated with Octominin-CNPs). Acclimatization was carried out for one week, maintaining the same rearing conditions. On the wound initiation day, anesthesia was carried out by submerging each fish in a 0.12% (*w*/*v*) tricaine solution, and the dermal wound of each fish was made between the anal and dorsal fins on the dark stripe of the left flank anterior position using a 3 mm diameter biopsy punch on day 0, except in the NC group. Each wounded fish was treated with the assigned treatment (20 μg/wound/fish) via a direct topical application on 0, 2, and 4 dpw, and held outside (on a moist gauze) at 25 ± 2 °C for a total of 4 min to increase treatment absorption. The NC group (no wound) was also kept outside for the same duration of time to provide the same conditions for the rest of the groups.

### 4.7. Morphological Wound Healing Efficacy Analysis of Octominin-CNPs

Five fish from each group were selected at the 2 dpw and reared in individual tanks (500 mL). Visual observations of the wound site were conducted by using a digital camera-equipped stereo microscope (Leica^®^ S8 APO, Leica Microsystems GmbH, Wetzlar, Germany), coupled with an LED-based light source (Leica^®^ KL300 LED, Leica Microsystems GmbH, Wetzlar, Germany) at 2, 4, 7, 14, and 21 dpw. Quantification of the images was performed using the ImageJ software to determine the WHR and WHP upon the treatment of Octominin-CNPs. The completion of the wound healing was assumed when there was no distinguishable wound area or if complete skin pigmentation was achieved. The following equations are derived to measure and analyze the WHR and WHP (%) of the same individual fish.

$$WHP(\%)=\frac{\left(Ao-At\right)}{Ao}\times 100$$

$$WHR=\frac{\left(Ao-At\right)}{dpw}$$

Ao—Area of the open wound at 0 dpw.

At—Area of the open wound at relevant time point (t).

### 4.8. Histological Analysis of the Wound Healing Activity of Octominin-CNPs

To further elaborate on the wound healing effect of Octominin-CNPs, H&E staining was conducted according to a previously published method [30]. Briefly, three fish from all five groups (7 dpw) were euthanized using a lethal dose of tricaine. The wounded muscle was surgically isolated and immediately immersed in PBS. Isolated muscles were fixed in a 10% neutral buffered formalin solution. Prior to sectioning, tissues were decalcified by immersing them in a 0.5 M EDTA (neutral pH) for 3 days at 28 °C. Then, the tissues were washed for 12 h in mildly free-flowing water to remove excess EDTA and formalin. Tissue dehydration was performed by submerging them in xylene and an ascending alcohol gradient in a Leica^®^ TP1020 Semi-enclosed Benchtop Tissue Processor (Leica Biosystems, Nussloch, Germany). The processed tissues were paraffin wax-embedded by implementing a tissue embedder (Leica^®^ EG1150 Tissue Embedding Center, Nussloch, Germany), prior to sectioning using a microtome (Leica^®^ RM2125 microtome, Nussloch, Germany). Slide preparation was performed using 4 μm thick tissue sections, and each slide went through a series of xylene and ethanol solutions during staining process done by the H&E staining kit (Abcam; Waltham, MA, USA) following the manufacturer’s guidelines. Stained slides were observed using a digital camera-attached microscope (LEICA^®^ DCF450-C, Leica Microsystems GmbH, Wetzlar, Germany).

### 4.9. mRNA Expression of the Wound Healing Marker Genes in Zebrafish

Six fish per group per time point (2 and 7 dpw) were euthanized with a lethal tricaine dose to isolate the wound muscle tissues. Isolated muscle tissues were snap-frozen in liquid nitrogen and divided into three replicates (two tissues/replicate). Total RNA was isolated using the TRIzol^®^ method, as mentioned in a previous publication [30]. Concentration of the isolated RNA was measured using a NanoDrop One spectrometer (Thermo Fisher Scientific, Madison, WI, USA), and cDNA preparation was performed using the PrimeScript 1st strand cDNA Synthesis Kit (TaKaRa Bio Inc., Shiga, Japan), following the manufacturer’s protocol. Dilution was conducted to prepare the final cDNA sample, which was stored at −20 °C. Relevant wound healing marker genes, as shown in Table S1, were selected, and qRT-PCR was performed using a Thermal Cycler Dice Real Time System (TaKaRa Bio Inc., Shiga, Japan). The relative expression of the mRNA levels of each gene was quantified using the 2^−ΔΔCt^ method [37], and zebrafish *β-actin* was used as the housekeeping gene.

### 4.10. Statistical Analysis

Data analysis was performed using the GraphPad Prism software version 8 (San Diego, CA, USA). A one-way analysis of variance (ANOVA) test was performed separately for cell viability and gene expression, while a two-way ANOVA test was performed for the cell migration assay and in vivo dermal wound healing experiment. To determine the significance between treatment groups, Dunnett’s multiple comparison test (for one-way ANOVA) and Tukey’s range test (for two-way ANOVA) were performed separately, and statistical significance was determined at *p* < 0.05. Data for replicates are expressed as means ± standard error of mean (SEM).

## 5. Conclusions

In the present study, Octominin was successfully encapsulated in CN to produce Octominin-CNPs with minimum toxicity in HDF cells and zebrafish larvae compared to Octominin. Furthermore, Octominin-CNPs accelerated wound healing in both in vitro and in vivo conditions, showing promise as an alternative therapeutic agent for dermal wound treatment. Encapsulation of Octominin into CN offers protection of the payload and enables a controlled, lasting release, thereby enhancing stability and reducing the loss by degradation, which is typically observed with AMPs. Moreover, Octominin-CNPs could be implemented in aquaculture for wound management owing to the combined natural wound healing properties of CN and antimicrobial activity of Octominin.

## Data Availability

The data presented in this study are available from the corresponding author upon reasonable request.

## Supplementary Materials

The following supporting information can be downloaded at: https://www.mdpi.com/article/10.3390/ijms26199701/s1.
