# Supplementary material for: Octopus minor Antimicrobial Peptide-Loaded Chitosan Nanoparticles Accelerate Dermal Wound Healing
Source: Int J Mol Sci. 2025 Oct 5;26(19):9701. doi: 10.3390/ijms26199701 (PMC12525058; doi:10.3390/ijms26199701)
Supplement: Supplementary file 1 [file ijms-26-09701-s001.zip › ijms-3866651-supplementary.pdf]

Supplementary Material:

Table S1. List of zebrafish genes and primers used for gene expression analysis.

Gene name	Tm (°C)	Primer name	Primer sequence (5′-3′)	Accession no.
Chemokine (C-X-C motif) ligand 18b (*cxcl18b*)	58	cxcl18b -F	CTGCTGCTCGCGGTAGTTTA	NM_001115060
		cxcl18b -R	TCAACTTTGTCGCAGTTTGG	
Chemokine (C-C motif) ligand 34a, duplicate 4 (*ccl34a4*)	58	ccl34a.4-F	TGCAGCTCAACCAGAAGATG	NM_001130655.1
		ccl34a.4-R	CTTTGACGCATGGAGGATTT	
Interleukin-1β (*il1β*)	58	il1β-F	TCAAACCCCAATCCACAGAG	AY340959.1
		il1β-R	TCACTTCACGCTCTTGGATG	
Interleukin-10 (*il10*)	58	il10-F	CCCTATGGATGTCACGTCATG	AY887900.1
		il10-R	CATATCCCGCTTGAGTTCCTG	
Tumor necrosis factor-α (*tnfα*)	58	tnfα-F	AGAAGGAGAGTTGCCTTTACCGCT	AY427649
		tnfα-R	AACACCCTCCATACACCCGACTTT	
Matrix metalloproteinase 9 (*mmp9*)	56	mmp9-F	TTTGCCCTGATCGTGGATAC	AY151254.1
		mmp9-R	GGGAAACCCTCCACGTATTT	
Matrix metalloproteinase 13 (*mmp13*)	57	mmp13-F	GAGAAGGTTTGGGCTCTCTATG	AF506756.1
		mmp19-F	TGAGTTGCTGTCTTCCTTGTAG	
Beta-actin (*β actin*)	58	β actin- F	AATCTTGCGGTATCCACGAGACCA	AF025305.1
		β actin- R	TCTCCTTCTGCATCCTGTCAGCAA	

Tm: melting temperature

A

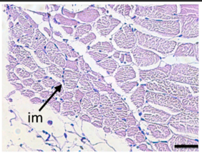

Inflammation
0. Absent
1. Mild (Inflammatory cellular infiltration into the wound tissue)
2. Moderate (Increased epidermal tissue spaces and loosened connective tissues)
3. High (Excessive tissue swelling)

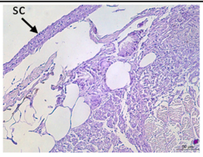

Scab
0. Absent
1. Mild (Healed or healing upper layer of the wound tissue)
2. Moderate (Separation of wound layer from the wound surface)
3. High (Free space from wound tissue and separated scab)

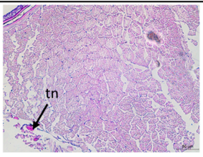

Tissue necrosis
0. Absent
1. Mild (Healed or healing upper layer of the wound tissue)
2. Moderate (Separation of wound layer from the wound surface)
3. High (Muscle fiber and structure damage)

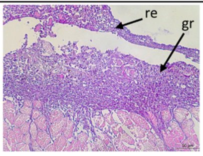

Tissue re-epithelialization and granulation
0. Absent
1. Mild (Tissue enlargement, collagen fiber deposition, and formation of new connective tissues)
2. Moderate (Granulation tissue enlargement and tissue thickness increasing)
3. High (Cell proliferation increased hyper-genesis)

B

Group	Inflammation (im)	Scab (sc)	Tissue necrosis (tn)	Re-epithelialization (re)	Granulation tissue (gr)
Vehicle	3.0 ± 0.0	1.5 ± 0.6	2.7 ± 0.3	3.0 ± 0.0	3.0 ± 0.0
CNPs	1.7 ± 0.6	2.3 ± 0.6	1.3 ± 1.0	2.3 ± 0.6	1.3 ± 1.0
Octominin	3.0 ± 0.0	2.7 ± 0.6	2.3 ± 0.6	3.0 ± 0.0	2.3 ± 0.6
Octominin+CNPs	2.3 ± 0.3	1.5 ± 0.6	1.3 ± 1.0	1.5 ± 0.6	2.3 ± 0.6

Figure S1. Semi-quantitative analysis of the hematoxylin and eosin (H&E) stained tissue samples (n=3/treatment). (**A**) Representative microscopic images used to describe the parameters of the semi-quantitative analysis (×200; scale bar = 50 µm) and the scoring criteria. (**B**) Mean score of each treatment group for each parameter from 0–3. Isolated wound muscle sections at 7 dpw were stained with hematoxylin and eosin (H&E) used in the evaluation. Data is expressed as Means ± SEM.
